# Convergent nitrogen–phosphorus scaling relationships in different plant organs along an elevational gradient

**DOI:** 10.1093/aobpla/plaa021

**Published:** 2020-05-25

**Authors:** Xiaoping Chen, Mantang Wang, Man Li, Jun Sun, Min Lyu, Quanlin Zhong, Dongliang Cheng

**Affiliations:** 1 Fujian Provincial Key Laboratory of Plant Ecophysiology, Fujian Normal University, Fuzhou, Fujian Province, China; 2 Key Laboratory of Humid Subtropical Eco-geographical Process, Ministry of Education, Fuzhou, Fujian Province, China; 3 School of City and Architecture Engineering, Zaozhuang University, Zaozhuang, Shandong Province, China

**Keywords:** Nutrient allocation, limiting nutrient, plant economics spectrum (PES), plant organ, subtropical forest, Wuyi Mountains

## Abstract

A general relationship between the nitrogen (N) and phosphorus (P) content of all plant organs (e.g. leaf, stem, and root) is hypothesized to exist according to whole-plant economics spectrum (PES) theory, but the evidence supporting these expected patterns remains scarce. We measured the N and P content of the leaves, twigs and fine roots of 64 species in three different forest communities along an elevational gradient (evergreen broad-leaved forest, 1319 m a.s.l., coniferous and broad-leaved mixed forest, 1697 m a.s.l., and deciduous forest, 1818 m a.s.l.) in the Wuyishan National Nature Reserve, southeastern China. The scaling relationship between the N and P content and the linear regression relationship between the N:P ratio and N and P content were analysed. The leaf N and P content was significantly higher at the high-elevation site than at the low- or middle-elevation sites (*P* < 0.001). The N and P content followed a power-law relationship with similar scaling slopes between organs. The N (common slope, 1.13) and P (common slope, 1.03) content isometrically covaried among leaves, twigs and roots. The scaling exponents of the N–P relationship were not significantly different from 1.0 in all organs, with a common slope of 1.08. The scaling constants of N–P decreased significantly (*P* < 0.05) from the highest value in fine roots (β = 1.25), followed by leaves (β = 1.17), to the lowest value in twigs (β = 0.88). Standardized major axis (SMA) analyses and comparisons of 95 % confidence intervals also showed that the numerical values of the scaling slopes and the scaling constants did not differ regardless of elevation. The N content, but not the P content, accounted for a large proportion of the variation in the N:P ratio in leaves (N:P and N: *r*^2^ = 0.31, *F* = 33.36, *P* < 0.001) and fine roots (N:P and N: *r*^2^ = 0.15, *F* = 10.65, *P* < 0.05). In contrast, the N:P ratio was significantly related to both the N and P content in the twigs (N:P and N: *r*^2^ = 0.20, *F* = 17.86, *P* < 0.001; N:P and P: *r*^2^ = 0.34, *F* = 35.03, *P* < 0.001, respectively). Our results indicate that different organs of subtropical woody plants share a similar isometric scaling relationship between their N and P content, providing partial support for the PES hypothesis. Moreover, the effects of the N and P content on the N:P ratio differ between metabolic organs (leaves and fine roots) and structural organs (twigs), elucidating the stoichiometric regulatory mechanism of different organs.

## Teaser

We found the scaling exponents of the N-P relationship were not significantly different from 1.0 in all organs, with a common slope of 1.08. The numerical values of the scaling slopes and the scaling constants did not differ regardless of elevation. These results indicated the different organs of subtropical woody plants share a similar isometric scaling relationship between their N and P content. Moreover, the effects of the N and P content on the N:P ratio differ between metabolic organs (leaves and fine roots) and structural organs (twigs), elucidating the stoichiometric regulatory mechanism of different organs.

## Introduction

Nitrogen (N) and phosphorus (P) are essential nutrients that influence many plant functions, such as growth, reproduction and defence (Li *et al.* 2010; Crous *et al.* 2017). Therefore, the allocation of N and P (content and stoichiometric ratio) has long been of interest in studies on the evolution of plant functional traits, as well as in studies on the material and energy cycles of terrestrial ecosystems (Ågren 2004; Elser *et al.* 2007; Heberling and Fridley 2012; Mo *et al.* 2019). Plants need to allocate N and P to different organs (i.e. leaves, twigs and roots) to meet multiple functional requirements. Given that the functional requirements for N and P might be organ-specific (Reich *et al.* 2008; Zhao *et al.* 2016), knowledge of nutrient partitioning among plant organs is critical to explain organ function, organ growth and turnover rates and plant growth form (Westoby *et al.* 2002; Kerkhoff *et al.* 2006; Minden and Kleyer 2014; Yang *et al.* 2014). Specifically, the N and P content in the different organs of a plant reflect nutrient uptake and utilization efficiency during plant growth (Wright and Westoby 2003), while the N–P relationship in tissues can aid in identifying the flows of energy and element cycling across plants, which can advance our understanding of ecological dynamics and processes (Reich *et al.* 2010).

As both elements are important for plant metabolism, the relationship between N and P among different plants or environments is of particular interest (Reich *et al.* 2010). The scaling relationship has widely been used to analyse the N and P partitioning in woody plants because it provides information on the relative allocation of nutrients in different organs across diverse plant species and has a high degree of predictive power (e.g. Reich *et al.* 2008). The scaling relationship between the N and P content of different organs can be described by the equation *y* = β*x*^α^, where *y* and *x* are the N and P content, respectively, β is the normalization constant, or scaling constant on log–log axes, and α is the scaling exponent, or scaling slope on log–log axes. Previous studies show that the convergent scaling relationship holds true in leaf N and P content throughout ecosystems and biomes owing to biophysical constraints and evolutionary selection (Wright *et al.* 2004; Reich *et al.* 2010). Although it has been suggested that the strategic allocation of N and P in plants may follow fundamental rules (Niklas *et al.* 2005; Kerkhoff *et al.* 2006; Niklas and Cobb 2006; Ågren 2008; Reich *et al.* 2010), there is no consensus on the scaling slopes of N and P relationships. For example, using comprehensive global data, Wright *et al.* (2004) and Reich *et al.* (2010) reported that the scaling slope of leaf N vs. leaf P was approximately 2/3, whereas Kerkhoff *et al.* (2006) stated that it was almost 3/4. Inconsistent scaling slopes in leaf N and P scaling relationships might be due to the different taxonomic groups and/or geographical locations being studied. For example, Tian *et al.* (2017) revealed large variations in the leaf N and P scaling slopes, which decreased from tropical to temperate to boreal zones.

N and P affect the metabolism and function of all plant organs, such as twigs and fine roots (Kerkhoff *et al.* 2006). An emerging body of evidence indicates that the characteristics of leaves (Wright *et al.* 2004), wood (Chave *et al.* 2009) and fine roots (Bergmann *et al.* 2017) are coordinated, often co-scaled across plant species, and constitute a whole-plant economics spectrum (PES) axis of slow vs. fast growth (e.g. Reich 2014). The PES suggests that fundamental constraints on fast and slow growth depend upon the coordination of different organs (Reich 2014). If plant N and P allocation strategies also depend on the PES, it is reasonable to suppose that a general scaling relationship between N and P content might exist for all organs, because nutrients and water are integrated across leaf, twig and root systems for their maximum fitness. This is particularly interesting if the convergent scaling relationship among different organs, including the scaling constant, shows the existence of concordant strategies among different species and organs. This concept is supported by studies on the N and P content in different organs. For example, in a broad survey of leaf, twig and root N and P content data, Kerkhoff *et al.* (2006) demonstrated that the 95 % confidence intervals (CIs) for the scaling slopes of P and N in different organs were coincident (see also Zhao *et al.* 2016). However, although Kerkhoff *et al.* (2006) collected data from a variety of plants, they did not collect nutrient data for all three organs. Therefore, global data on the N and P content of different organs, collected from studies in which different survey methodologies and diverse species catalogues are used, may fail to capture general N and P scaling relationships between organs. Furthermore, the N and P scaling relationships of plant organs could shift in response to environmental variation and the plant’s nutritional requirements (Reich *et al.* 2008). It remains unclear whether a general N and P scaling relationship exists for all organs.

Another important factor that affects plant growth is the N:P ratio in different organs (Koerselman 1996; Güsewell 2004; Elser *et al.* 2007). For example, the growth rate hypothesis states that a relatively large amount of P-rich RNA is needed to support rapid rates of protein synthesis (Elser *et al.* 1996; Sterner and Schulz 1998). Consequently, the elemental stoichiometry of fast-growing tissues or individuals is tipped towards P, so fast-growing organisms have a low tissue N:P ratio. Obviously, the leaf N:P ratio is determined by the leaf N and P content but, based on global leaf nutrient data, Reich and Oleksyn (2004) concluded that the leaf N:P ratio near the equator (i.e. tropical regions) was more strongly affected by variation in P compared with variation in N, while at higher latitudes (i.e. temperate regions) it was N-controlled, due to mean air temperature and soil characteristics. In contrast, Han *et al.* (2005) showed that the mean N:P mass ratio of China’s flora (14.4) was larger than the global averages reported by Elser *et al.* (2000) (11.0) and Reich and Oleksyn (2004) (11.8) because of a lower leaf P content. Given the functional coordination of above- and below-ground organs (Yuan *et al.* 2011; Bergmann *et al.* 2017), it is unclear whether similar high N:P ratios exist in the twigs and fine roots of Chinese subtropical plants, nor how the N and/or P content of twigs and fine roots influence the N:P ratio.

In this study, we aim to determine whether the general relationship exists that describes the partitioning of N and P allocation within and among the major organs of woody plants, and whether these associations can be generalized across plant communities. Specifically, we ask two questions: how are the N and P content allocated among leaves, twigs and fine roots across species, and how do the N and/or P content of leaves, twigs and fine roots influence the N:P ratio? Our specific hypotheses are (i) that the scaling relationship between the N and P content represents a fundamental biological limitation for growth and it should be coordinated in a variety of organs, so the scaling slopes between N and P content will be similar for leaves, twigs and roots; (ii) the constants of such scaling relationships differ, reflecting differing nutrient requirements between organs; and (iii) for leaves, twigs and fine roots, the patterns of the N and/or P content influencing the N:P ratio will be similar. We investigate what the nutrient allocation strategies of 64 species are among different plant functional organs in three forest communities (evergreen broad-leaved forest, coniferous and broad-leaved mixed forest and deciduous forest) at different elevations (1319, 1697 and 1818 m above sea level (a.s.l.)) in the Wuyi Mountains, southeast China.

## Methods

### Study site

The study was conducted in the Wuyishan National Nature Reserve, Jiangxi, southeastern China (27°4811″–28°00′35″N, 117°39′30″–117°55′47″E, 2157.7 m a.s.l.). The reserve has a typical mid-subtropical monsoon climate. The annual precipitation averages 2583 mm, most of which falls in the rainy season that occurs between April and June. The mean annual air temperature decreases from 13 °C at 1200 m a.s.l. to 11 °C at 1600 m a.s.l., and 9 °C at 2000 m a.s.l.; the maximum and minimum air temperatures occur in July and January, respectively. The mean annual relative humidity is 72 to 92 % (Lin *et al.* 1982). The soil texture is mainly a haplic lixisol, commonly formed from sandstone, with variable soil depths (Chen 2000). The Wuyishan National Nature Reserve has a typical elevational vegetation gradient, from evergreen broad-leaf forests at low elevations to mountainous steppes at high elevations, influenced by the climate.

### Plant and soil sampling

In August 2016, sites in three forest types (evergreen broad-leaved forest (EF, 1319 m a.s.l.), coniferous and broad-leaved mixed forest (MF, 1697 m a.s.l.) and deciduous forest (DF, 1818 m a.s.l.)) in the Wuyishan National Nature Reserve were used to test the relationship between the N and P content allocation in woody plants. Three 20 × 20 m plots were established in each of the three forest types; all plots were located at least 20 m apart. Information on stand density, stem diameter at breast height and plant height within each of the forest types is presented in Table 1. The dominant species in the EF were *Rhododendron simiarum*, *Schima superba*, *Cyclobalanopsis glauca* and *R. ovatum*. The dominant species in the MF were *R. simiarum*, *Tsuga chinensis*, *C. glauca* and *Illicium minwanense*. The DF was dominated by *I. minwanense*, *Symplocos paniculata*, *Eurya saxicola* and *Padus racemosa*. A total of 64 species were sampled across the three forest types, representing 42 genera and 26 families. Three species were common to all three communities and six species were common to two forest types **[see **Supporting Information—**Table S1****]**. The same species in different forest types were treated as different species. Three individuals of each species were sampled.

**Table 1.** Description of the three communities at different elevations (mean ± standard error). Different letters in a column indicate that significant differences existed between elevations (*P* < 0. 05). EF, evergreen broad-leaved forest; MF, coniferous and broad-leaved mixed forest; DF, deciduous forest; STN, soil total nitrogen content; STP, soil total phosphorus content.

Vegetation type	Elevation zone	Stand density	Mean DBH	Mean height	STN	STP
		(trees/hm^2^)	(cm)	(m)	(mg/g)	(mg/g)
EF	Low (1319 m)	3033. 33 ± 200 **a**	13. 77 ± 1. 46 **b**	7. 87 ± 0. 07 **b**	4. 84 ± 0. 04 **a**	0. 46 ± 0. 01 **b**
MF	Middle (1697 m)	1133. 33 ± 164. 15 **b**	21. 39 ± 0. 8 **a**	10. 56 ± 0. 21 **a**	5. 25 ± 0. 27 **a**	0. 38 ± 0. 02 **c**
DF	High (1818 m)	2725 ± 163. 94 **a**	11. 47 ± 0. 67 **bc**	6. 94 ± 0. 24 **b**	6. 05 ± 0. 22 **a**	0. 65 ± 0. 01 **a**

For each individual, three branches with their tips at the outer surface of the plant’s crown were randomly chosen. The first branch-point from the growing tip was located. Twigs and leaves were selected from the terminal branches of the current-year shoots. The sampled leaves had not lost any part of their area to herbivory and were not damaged in any other way. They had expanded to over 50 % of their typical final length and were close to median size. From each selected twig, fully expanded leaves (*n* = 225) and twigs (*n* = 225) were harvested.

We collected fine roots (≤2-mm diameter) from the top 30 cm of the soil layer along the tap roots from the same three individuals of each species that were sampled for leaves and twigs. The fine roots were taken to the laboratory and immediately a sieve (0.15-mm pore size) was used to wash the fine roots with fresh water, in order to remove soil particles and organic debris. The fine roots were then separated into live and dead roots according to morphological criteria. Live fine roots that were intact, tough and flexible were analysed (*n* = 186).

Soil samples from the 0–10 and 10–20 cm soil layers, taken at three randomly selected locations with low levels of interference in each plot, were mixed thoroughly and roots and litter were removed.

### Measurement of N and P content

The leaf, twig and fine root samples were dried at 75 °C for 48 h, while the soil samples were naturally air-dried. After drying, the samples were ground to a powder with a grinder and then passed through a 100-mesh sieve (0.15 mm). The plant N content was determined using a Vario EL III elemental analyser (Elementar, Germany), and the soil N content was determined using a Vario Max elemental analyser (Elementar, Germany). Each powder sample was digested with H_2_SO_4_–HClO_4_ (4:1, v:v) at 300 °C for 3–4 h until the solution became clear (Sommers and Nelson 1972, Hidaka and Kitayama 2011). The digests were diluted to 100 mL with deionized water. The P content was measured using the molybdate/ascorbic acid method and a continuous flow analyser (Skalar SAN++, The Netherlands). The total soil N and P content of each forest type is shown in Table 1.

### Scaling analysis

To evaluate whether the N and P content and their ratio were significantly different across organs along an elevational gradient, traits were compared using one-way analysis of variance in the agricolae package in R (v.3.2.3; R Foundation for Statistical Computing, Vienna, Austria). A scaling approach that consisted of *y* = β*x*^α^, where *y* and *x* are N and P content, respectively, β is the normalization constant, and α is the scaling exponent, was used to examine covariations in N and P concentrations. When α = 1, the equation describes an isometric relationship, and when α ≠ 1, the equation describes an allometric relationship. To minimize the sum of squared residuals, the conventional practice in allometric studies is logarithmic transformation. The log–log scaling relationship log *y* = log β + α log *x* and a common scaling slope in which observations of different organ groups with no statistically significant differences in the numerical values of α were quantified with standardized major axis (SMA) slopes (Warton *et al.* 2012) using the smatr package 3.4-3 in R. The smatr package was used to provide the model type II equivalent of ordinary least squares (OLS) standard analyses of covariance (Warton *et al.* 2006, 2012), using a likelihood ratio test for the common slope and comparing it to a chi-squared distribution (Warton *et al.* 2006). The significance level for testing slope heterogeneity across organs or the elevational gradient was set at *P* < 0.05 (e.g. slope heterogeneity was rejected if *P* > 0.05). Data showing no statistically significant differences in the numerical values of α were then examined to determine a common scaling slope. The common slope estimate was obtained from a pooled variance/covariance matrix. The N and P content and their ratio in different organs were log-transformed before analysis to increase normality. Pearson’s correlation coefficient was used to quantify the degree of association between the N:P ratio and N, and between the N:P ratio and P. If correlations were significant (*P* < 0.05), the relationships between the N:P ratio and N and between the N:P ratio and P were described by a linear regression function in R.

## Results

### Organ and soil nutrients (and their relationships) along an elevational gradient

**Figure 1.**
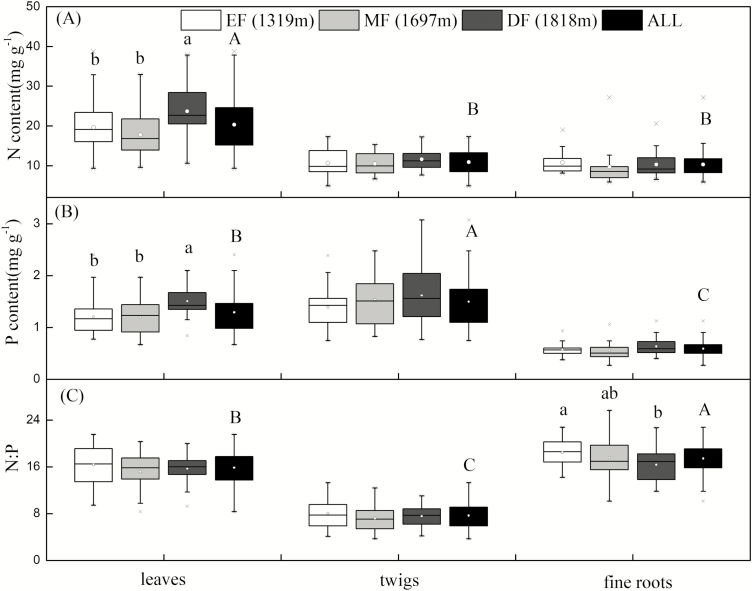
Comparisons of (A) nitrogen (N) content, (B) phosphorus (P) content and (C) the N:P ratio of leaves, twigs and fine roots in an evergreen broad-leaved forest (EF), a coniferous and broad-leaved mixed forest (MF), a deciduous forest (DF) and all elevations (ALL). The distribution of N, P and N:P for different plant organs is characterized by the box plots, where the box length provides the interquartile range, the bottom and the top of the box represent the 25th and 75th percentile, respectively, the horizontal line within the box represents the median value, and the circle within the box represents the mean value. The crosses denote values outside the whisker limit. Different lower- and upper-case letters above the columns indicate significant differences (*P* < 0.05) between elevations and organs, respectively.

The N and P content differed significantly among organs (Fig. 1). Specifically, N was significantly higher in the leaves (20.32 ± 0.77 mg g^-1^) than in the other organs (*P* < 0.001; Fig. 1A). The P content was significantly higher in the twigs (1.50 ± 0.05 mg g^-1^, *P* < 0.001), compared with the leaves (1.29 ± 0.04 mg g^-1^, *P* < 0.001) or fine roots (0.59 ± 0.02 mg g^-1^, *P* < 0.001; Fig. 1B). In contrast, the N:P ratio was the highest in the fine roots (17.42 ± 0.23, *P* < 0.001) and lowest in the twigs (7.67 ± 0.26, *P* < 0.001; Fig. 1C).

The leaf N and P content were significantly higher at the high-elevation site than either the low- or middle-elevation sites (*P* < 0.001; Fig. 1A and B), whereas no significant differences in N or P along the elevations were observed for the other organs. The N:P ratio only in the fine roots was significantly lower at our high-elevation site than either of our lower elevation sites (*P* < 0.001; Fig. 1C).

Soil N had no significant difference along an elevational gradient (*P* > 0.05; Table 1), while soil P was highest at the high-elevation site (0.65 ± 0.01 mg g^-1^, *P* < 0.001; Table 1) and lowest at the middle-elevation site (0.38 ± 0.02 mg g^-1^, *P* < 0.001; Table 1). There were no significant relationships between soil N content and organ nutrients (*P* > 0.05; Table 2). In contrast, leaf N and fine root P were significantly positively correlated with soil P (*P* < 0.05; Table 2).

**Table 2.** Bivariate relationships between soil nitrogen (N) and phosphorus (P) content and plant nutrient content along an elevational gradient. * indicates a significant correlation at the 0.05 level (*P* < 0.05); ** indicates a significant correlation at the 0.01 level (*P* < 0. 01).

	N (mg g^-1^)			P (mg g^-1^)			N:P		
Nutrient	leaves	twigs	fine roots	leaves	twigs	fine roots	leaves	twigs	fine roots
N_soils_ (mg g^-1^)	0.80	0.89	-0.28	0.93	0.94	0.84	-0.40	-0.41	-0.99
P_soils_ (mg g^-1^)	1.00^**^	0.99	0.34	0.96	0.55	0.99*	0.22	0.21	-0.75

### Effects of organ and elevation on the scaling relationship between N and P

**Figure 2.**
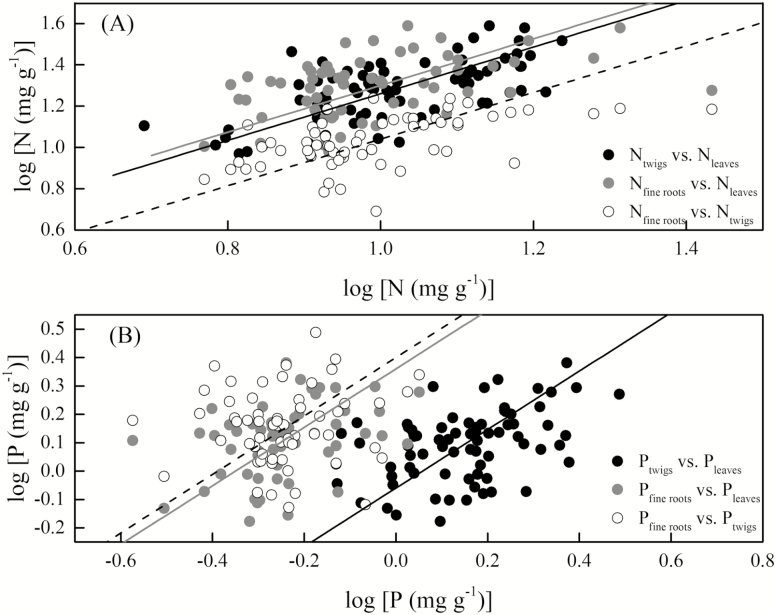
Scaling of nitrogen (N) and phosphorus (P) content (% dry mass) among leaves, twigs and fine roots across all 64 species. (A) N covariance among the leaves, twigs and fine roots (the common slope was α = 1.13, 95 % CI = 1.00–1. 28, *P* = 0.26). (B) P covariance among the leaves, twigs and fine roots (the common slope was α = 1.03, 95 % CI = 0. 90–1.18, *P* = 0.48). All lines are significant standardized major axis (SMA) regressions (*P* < 0. 05); where three lines are present indicating heterogeneity in the intercept, the regression lines for leaves vs. twigs are black, those for leaves vs. fine roots are grey and those for twigs vs. fine roots are dashed.

N covaried among the leaves, twigs and fine roots of the 64 species (the common slope was α = 1.13, 95 % CI = 1.00–1.28, *P* = 0.26; Fig. 2A). A similar relationship was observed for P (the common slope was α = 1.03, 95 % CI = 0.90–1.18, *P* = 0.48; Fig. 2B). The scaling slopes were all indistinguishable from 1, indicating an isometric relationship (*P* > 0.05; Fig. 2).

**Figure 3.**
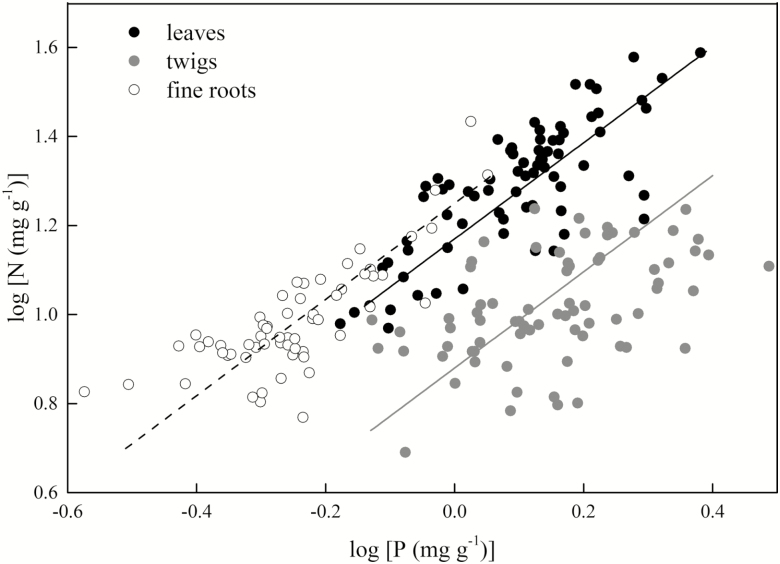
Scaling of nitrogen (N) and phosphorus (P) content (% dry mass) in leaves, twigs and fine roots across all 64 species. All lines are significant standardized major axis (SMA) regressions (*P* < 0.05); where three lines are present indicating heterogeneity in the intercept, the regression lines for leaves are black, those for twigs are grey, and those for fine roots are dashed. The scaling slopes of N and P in the different organs across all species did not differ significantly, with a common slope of 1.08 (95 % CI = 0. 98–1.19, *P* = 0. 10).

**Figure 4.**
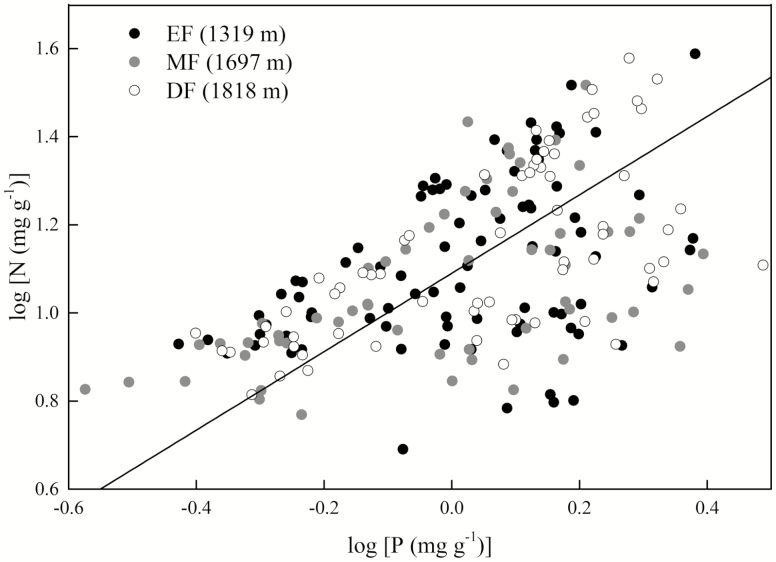
Effect of elevation on the scaling relationship between nitrogen (N) and phosphorus (P) content (% dry mass) in all 64 species. Lines are significant standardized major axis (SMA) regressions (*P* < 0. 05). The scaling slopes of N and P in the organs along an elevational gradient did not differ significantly, with a common slope of 0.89 (95 % CI = 0.79–1.00, *P* = 0.28) and with a common constant of 1.09 (95 % CI = 1.06–1.11, *P* = 0.84).

The N content was positively correlated with the P content in all organs (*P* < 0.001; Table 3). The scaling slopes of N and P in the different organs across all species did not differ significantly, with a common slope of 1.08 (95 % CI = 0.98–1.19, *P* = 0.10; Table 3; Fig. 3). However, the scaling constants of N and P differed significantly and decreased from the fine roots (β = 1.25) to the leaves (β = 1.17) to the twigs (β = 0.88) (*P* < 0.05; Fig. 3). The scaling slopes of N and P in all organs along an elevational gradient did not differ significantly, with a common slope of 0.89 (95 % CI = 0.79–1.00, *P* = 0.28) and with a common constant of 1.09 (95 % CI = 1.06–1.11, *P* = 0.84; Fig. 4).

**Table 3.** Summary of nitrogen–phosphorus (N-P) standardized major axis (SMA) regression results within organs and across species among three different plant communities. EF, evergreen broad-leaved forest; MF, coniferous and broad-leaved mixed forest; DF, deciduous forest.

N vs. P	slope	95 % CIs	constant	*R* ^2^	*P*	*n*
*Leaves*	1.20	1. 04–1.39	1.17	0.60	<0.001	75
EF	1.27	1. 01–1.69	1.19	0.60	<0.001	33
MF	1.07	0. 75–1.52	1.16	0.48	<0.001	20
DF	1.42	1. 07–1.88	1.12	0.62	<0.001	22
*Twigs*	0.91	0. 75–1.12	0.88	0.23	<0.001	75
EF	1.13	0. 81–1.58	0.86	0.13	<0.05	33
MF	0.83	0. 54–1.25	0.87	0.25	<0.05	20
DF	0.71	0. 50–0.99	0.92	0.45	<0.001	22
*Fine roots*	1.05	0. 90–1.23	1.25	0.63	<0.001	61
EF	1.06	0. 78–1.45	1.25	0.69	<0.001	20
MF	1.08	0. 82–1.41	1.28	0.61	<0.001	19
DF	1.05	0. 80–1.38	1.22	0.65	<0.001	22

### Relationships between the N and P content and the N:P ratio within organs

**Figure 5.**
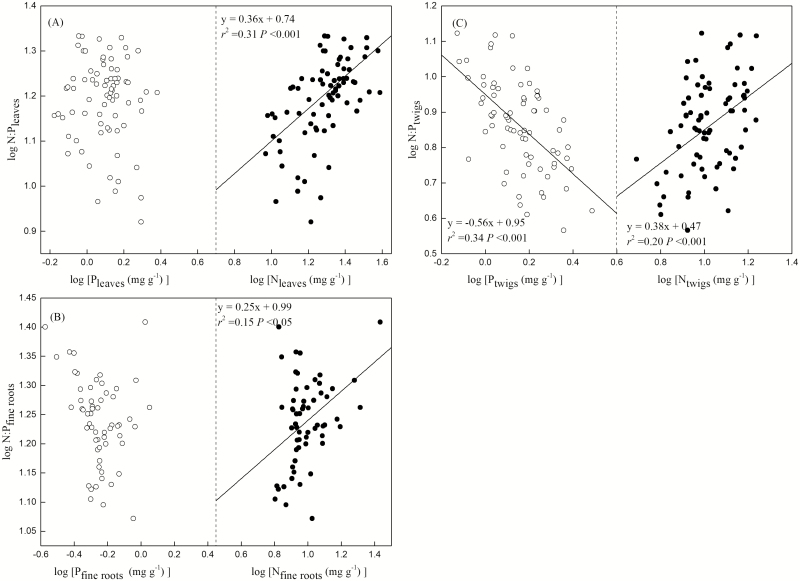
Relationships between the nitrogen (N) and phosphorus (P) content of leaves, twigs, and fine roots and the N:P ratio. All lines are significant regressions (*P* < 0. 05).

In the leaves and fine roots, the N:P ratio was significantly correlated with the N content but not with the P content (Fig. 5A and B; see **Supporting Information—Table S2**). Specifically, the N content accounted for a large proportion of the variation in the N:P ratio in the leaves (*r*^2^ = 0.31, *F* = 33.36, *P* < 0.001; Fig. 5A; **see ****Supporting Information—Table S2**) and fine roots (*r*^2^ = 0.15, *F* = 10.65, *P* < 0.05; Fig. 5B). Across species, the N:P ratio was positively correlated with the N content and negatively correlated with the P content in twigs (N:P and N: *r*^2^ = 0.20, *F* = 17.86, *P* < 0.001; N:P and P: *r*^2^ = 0.34, *F* = 35.03, *P* < 0.001; Fig. 5C; **see ****Supporting Information—Table S2**).

## Discussion

Based on data from 64 woody species from a mid-subtropical mountain region, we assessed whether general scaling relationships between the N and P content existed for twigs and fine roots along an elevational gradient and, if they did, whether they were similar to those for leaves. Across species, the N and P content covaried in leaves, twigs and roots along an elevational gradient with a nearly isometric common scaling slope. This result highlights the coordinated nutrient-use strategies of all organs, as predicted by PES theory. The N:P ratio in the different plant organs was dependent on whether the organs were metabolically active (i.e. leaves and fine roots) or structural (i.e. twigs), indicating that an organ-specific pattern existed.

### Organ-specific N and P content

Consistent with the results of previous studies (e.g. Kerkhoof *et al.* 2006), our results showed that the N content of leaves was higher than that of twigs and fine roots, probably because plants preferentially allocated more N for metabolic and photosynthetic activity (Fig. 1). In contrast, the P content was significantly higher in twigs than in leaves and fine roots (Fig. 1). This may arise because plants require higher P investments in twigs for photosynthate loading of leaves, especially in P controlled environments (Marschner and Marschner 2012). Yu *et al.* (2018) found a decrease in soil total P concentration over the past 60 years across subtropical China, supporting the strong P control in these ecosystems. The total soil P content in our experiment sites was not significantly different from the total soil P content in subtropical China (Yu *et al.* 2018) (Table 1), but it was lower compared with the whole of China data set (Han *et al.* 2005). Therefore, it is possible that P could control plant growth in our experimental sites. The leaf P, but not the N content, (Fig. 1) was lower than that of 1251 terrestrial plant species across the world (i.e. the world-wide leaf P content: 1.42 ± 1.12 mg g^−1^, the world-wide leaf N content: 18.3 ± 8.71 mg g^−1^, Reich and Oleksyn 2004). However, the P content of twigs in our study (1.50 ± 0.05 mg g^−1^; Fig. 1) was higher than that reported by Kerkhoff *et al.* (2006) (0.89 ± 0.10 mg g^−1^) for 1287 species in 152 seed plant families. Twigs were selected from the current year’s branches in the present study, so the nutrient flow was likely to be relatively high. Therefore, the P content in the twigs increased and even exceeded the leaf P content. This suggests that twigs store more P in order to sustain leaves when P is limited. Taken together, these observations indicate that the organ with the highest N content is the leaf and the organ with the highest P content is the twig for subtropical plants in Wuyi Mountain (Fig. 1).

The N and P content differed significantly among different communities in leaves but not in twigs or fine roots (Fig. 1). Specifically, woody plants in EF and MF had lower leaf N and P content than those in DF (Fig. 1), suggesting that evergreen plants (EF and MF) employ a conservative strategy and invest proportionally more into leaf formation than into other organs (Poorter *et al.* 2010). In contrast, plants in DF may follow an expensive strategy with high leaf N and P content in order to promote rapid carbon gain (Wright *et al.* 2004). This is linked to rapid leaf turnover via abscission, which maintains relatively high rates of nutrient cycling (Santiago and Wright 2007; Finegan *et al.* 2015).

The leaf N and P content of 64 species were higher at the high-elevation site than either the low- or middle-elevation sites (Fig. 1). The differences in leaf P along an elevation gradient were not driven by soil because there was no significant relationship between the two variables. There might be a link with temperature, which is the primary limiting factor for plant growth along an elevational gradient; dynamic leaf P processes are very sensitive to temperature changes. Reich and Oleksyn (2004) found temperature-driven leaf P changes followed a hump-shaped relationship, with P decreasing with temperature from the tropics to the mid-latitudes. Higher temperatures lead to longer growing seasons and slower growth rates during the growing season and, hence, to lower P content in plants growing at low elevations compared with those growing at high elevations. Indeed, many studies have found that P content is significantly affected by elevation (Köhler *et al.* 2006; Soethe and Lehmann 2008; Macek *et al.* 2009; Sundqvist *et al.* 2011). In addition, we observed a significant relationship between soil P and leaf N, which indicated that plants growing at the high elevation site had an excessive N uptake in order to increase P uptake (Ågren 2008), resulting in the highest leaf N this site.

### Convergent N and P scaling relationships in leaves, twigs and fine roots

Consistent with the prediction of PES theory, our results showed that a convergent scaling relationship between N and P existed in the leaves, twigs and fine roots. This indicated that the nutrient-use strategies of all organs were remarkably consistent across species in subtropical mountain woody plants (Figs 2 and 3). In addition, the numerical values of the scaling exponents for N and P did not differ regardless of elevation, when not distinguishing between organs, and they even shared the common constant (Fig. 4). Consequently, the results of both the present study and previous studies demonstrate that the PES is applicable for water-related as well as carbon- and nutrient-related traits across scales (Reich 2014).

Reich *et al.* (2008) analysed a database of 2510 measurements taken from 287 species and reported strong respiration–N scaling relationships in all observations and for data averaged by species. They also reported that no consistent differences in the slopes of these log–log scaling relationships were observed among organs or among plant groups. Freschet *et al.* (2010) studied plant resource economics in relation to interspecific variations in the traits of different plant organs, specifically those traits that corresponded to plant defence and growth, and found them to be consistent across species’ organs in subarctic flora. In the present study, we found that the N and P content of the plant organs were related to each other in similar and predictable ways across species, independent of the environmental conditions which explained the individual nutrient allocation strategies and community assemblage processes (Table 2; Figs 2 and 3). Thus, it is reasonable to deduce that elevation and its associated abiotic factors such as temperature are not responsible for numerical differences among the scaling exponent. Evolution and biophysics both impose trade-off constraints (Reich *et al.* 1999) and drive multiple resource acquisitions that need to be coupled and linked among organs (Diáz *et al.* 1998) to maximize plant performance. This leads to a convergent scaling relationship of N and P content among organs.

However, in contrast with previously reported observations that N increases rapidly with P in particular organs (Wright *et al.* 2004; Niklas *et al.* 2005; Reich *et al.* 2010), our results indicate that an isometric relationship between N and P exists among organs (Fig. 3). Two phenomena could explain such a difference in results. Firstly, the subtropical region of China has experienced high levels of N deposition (30–37 kg ha^−1^ year^−1^; Jia *et al.* 2014; Zhang *et al.* 2017), which may have increased the plant N content (Peñuelas *et al.* 2015). Secondly, as noted previously, P, but not N, has long been considered the main factor that limits plant growth in subtropical regions (Han *et al.* 2005; He *et al.* 2008). It is probable that in order to increase P uptake, excessive N uptake occurs in all organs, which can drastically modify the N and P relationship and shift the scaling slopes (Ågren 2008). The highly significant relationship of soil P and leaf N supports this possibility (Table 2). Taken together, the scaling relationships of P and N in all organs were isometric in our study.

### Differences in the N:P ratio between metabolic and structural organs

Because of the isometric N and P relationship in all organs, the scaling constants of N and P (i.e. β ≈ N/P when α ≈ 1.0) and N:P were positively correlated and approximately equal. Therefore, the N:P ratio played an essential role in determining the N and P scaling relationship and it is possible that the relative growth of the entire organism can be predicted using only the leaves or another plant organ (Niklas *et al.* 2005).

We found that the N:P ratio in the leaves was 15.84 ± 0.36 (Fig. 1), which was consistent with those reported by Han *et al.* (2005) (15.0) for tree species and He *et al.* (2008) (15.9 ± 5.19, mean ± SD) for woody plants in China. Our results indicated that the N:P ratio in leaves and fine roots was affected more by the N content than the P content, whereas the N:P ratio in twigs was controlled by both the N and P content (Fig. 5; **see ****Supporting Information—Table S2**). In the leaves, N plays a major role in the synthesis of proteins that regulate photosynthetic and respiratory processes (Field and Mooney 1986), while only a tiny proportion of total P is incorporated into the photosynthetic apparatus (Veneklaas *et al.* 2012). Fine root traits are often considered analogs of leaf traits with respect to the PES because they are both metabolically active organs (Bergmann *et al.* 2017). Leaf function depends upon the nutrients absorbed by fine roots, while fine root growth, in turn, depends upon the carbohydrates produced by the leaves. Twigs are essential intermediaries that link leaves and fine roots and are responsible for supporting them by facilitating the transport of nutrients and water (Schreeg *et al.* 2014; Yan *et al.* 2016). Consequently, they require high N and P investment and high rates of N and P cycling in order to support high rates of photosynthate transportation and photosynthetic activity (Marschner *et al.* 1996).

Overall, leaf and fine root N content may play a pivotal role in determining the N:P ratio, while the N:P ratio in twigs was both N- and P-influenced. Consequently, our results indicate that metabolic organs (leaves and fine roots) and structural organs (twigs) have different stoichiometric regulatory mechanisms.

## Conclusion

In summary, heterogeneous environmental conditions and the various trade-off strategies caused different N and P content for leaves and a different N:P ratio for fine roots along an elevational gradient. A general isometric scaling relationship between the N and P content was shared by the three organs of woody plants from the Wuyi Mountains, supporting the PES theory. We also found that the N:P ratio in leaves and fine roots was controlled more by the N content than the P content, while the twig N:P ratio was both N- and P-controlled in subtropical mountain plants, suggesting that stoichiometric regulatory mechanisms varied among different functional organs (metabolic vs. structural). Considering that the mycorrhizal type and level of inoculation can influence nutrient acquisition (Bever *et al.* 2010; Poisot *et al.* 2011; Shi *et al.* 2012), the effect of mycorrhiza on plant functional traits should be investigated in future research. Because of recent rapid changes in vegetation, future studies on nutrient allocation, as well as on the traits of different organs, should take into consideration N and P limitation in different organs in the subtropical region, which has long been considered P-controlled.

## Supporting Information

The following additional information is available in the online version of this article—

**Table S1**. Excel spreadsheet of the raw data for all variables measured in the experiment.

**Table S2**. Bivariate relationships between N and P content and their ratio in leaf, stem, and root along an elevational gradient.

plaa021_suppl_Supplementary_Material

plaa021_suppl_Supplementary_Table_S1
