# Supplementary material for: Convergent nitrogen–phosphorus scaling relationships in different plant organs along an elevational gradient
Source: AoB Plants. 2020 May 25;12(3):plaa021. doi: 10.1093/aobpla/plaa021 (PMC7281873; doi:10.1093/aobpla/plaa021)
Supplement: plaa021_suppl_Supplementary_Material [file plaa021_suppl_supplementary_material.doc]

**Table S2.** Bivariate relationships between N and P contents and their ratio in leaf, stem and fine root along an elevational gradient.

| Forest type (elevation) | Trait | N_Leaves_ | P_Leaves_ | N_Stems_ | P_Stems_ | N_fine roots_ | P_fine roots_ | N:P_leaves_ | N:P_stems_ |
| --- | --- | --- | --- | --- | --- | --- | --- | --- | --- |
| EF  (1,319 m) | P_Leaves_ | 0.78^**^ |  |  |  |  |  |  |  |
|  | N_Stems_ | 0.69^**^ | 0.65^**^ |  |  |  |  |  |  |
|  | P_Stems_ | 0.30 | 0.51^**^ | 0.37^*^ |  |  |  |  |  |
|  | N_fine roots_ | 0.28 | 0.14 | 0.53^*^ | 0.03 |  |  |  |  |
|  | P_fine roots_ | 0.36 | 0.22 | 0.39 | -0.23 | 0.88^**^ |  |  |  |
|  | N:P_leaves_ | 0.55^**^ | -0.07 | 0.27 | -0.23 | 0.29 | 0.27 |  |  |
|  | N:P_stems_ | 0.44^*^ | 0.18 | 0.61^**^ | -0.48^**^ | 0.55^*^ | 0.61^**^ | 0.52^**^ |  |
|  | N:P_fine roots_ | 0.04 | 0.002 | 0.33 | 0.08 | 0.36 | -0.11 | 0.12 | 0.24 |
| MF  (1,697 m) | P_Leaves_ | 0.58^**^ |  |  |  |  |  |  |  |
|  | N_Stems_ | 0.66^**^ | 0.58^**^ |  |  |  |  |  |  |
|  | P_Stems_ | 0.29 | 0.46^*^ | 0.47^*^ |  |  |  |  |  |
|  | N_fine roots_ | 0.36 | 0.28 | 0.62^**^ | 0.28 |  |  |  |  |
|  | P_fine roots_ | 0.43 | 0.41 | 0.50^*^ | 0.22 | 0.85^**^ |  |  |  |
|  | N:P_leaves_ | 0.58^**^ | -0.32 | 0.20 | -0.08 | 0.12 | 0.1 |  |  |
|  | N:P_stems_ | 0.27 | 0.12 | 0.36 | -0.61^**^ | 0.25 | 0.22 | 0.18 |  |
|  | N:P_fine roots_ | 0.09 | -0.03 | 0.47^*^ | 0.22 | 0.44 | -0.07 | 0.10 | 0.19 |
| DF  (1,818 m) | P_Leaves_ | 0.80^**^ |  |  |  |  |  |  |  |
|  | N_Stems_ | 0.43^*^ | 0.41 |  |  |  |  |  |  |
|  | P_Stems_ | 0.13 | 0.4 | 0.63^**^ |  |  |  |  |  |
|  | N_fine roots_ | 0.57^**^ | 0.38 | 0.45^*^ | 0.26 |  |  |  |  |
|  | P_fine roots_ | 0.51^*^ | 0.37 | 0.21 | 0.13 | 0.86^**^ |  |  |  |
|  | N:P_leaves_ | 0.68^**^ | 0.12 | 0.15 | -0.27 | 0.45^*^ | 0.40 |  |  |
|  | N:P_stems_ | 0.13 | -0.18 | 0.03 | -0.70^**^ | 0.10 | 0.01 | 0.38 |  |
|  | N:P_fine roots_ | 0.22 | 0.12 | 0.52^*^ | 0.28 | 0.32 | -0.18 | 0.15 | 0.18 |
| ALL | P_Leaves_ | 0.76^**^ |  |  |  |  |  |  |  |
|  | N_Stems_ | 0.62^**^ | 0.58^**^ |  |  |  |  |  |  |
|  | P_Stems_ | 0.25^*^ | 0.47^**^ | 0.47^**^ |  |  |  |  |  |
|  | N_fine roots_ | 0.38^**^ | 0.25 | 0.52^**^ | 0.2 |  |  |  |  |
|  | P_fine roots_ | 0.45^**^ | 0.37^**^ | 0.37^**^ | 0.12 | 0.84^**^ |  |  |  |
|  | N:P_leaves_ | 0.55^**^ | -0.1 | 0.22 | -0.21 | 0.27^*^ | 0.23 |  |  |
|  | N:P_stems_ | 0.31^**^ | 0.08 | 0.42^**^ | -0.55^**^ | 0.31^*^ | 0.24 | 0.41^**^ |  |
|  | N:P_fine roots_ | 0.06 | -0.05 | 0.37^**^ | 0.12 | 0.38^**^ | -0.15 | 0.14 | 0.23 |

Note: EF, evergreen broad-leaved forest; MF, coniferous and broad-leaved mixed forest; DF, deciduous forest; ALL, all forest types (elevation). * indicates a significant correlation at the 0.05 level (*P* < 0. 05); * * indicates a significant correlation at the 0.01 level (*P* < 0. 01).
